# Identification of Immunodominant Outer Membrane Proteins of *Fusobacterium necrophorum* from Severe Ovine Footrot By MALDI-TOF Mass Spectrometry

**DOI:** 10.1007/s00284-021-02383-2

**Published:** 2021-02-27

**Authors:** S. Farooq, S. A. Wani, S. Qureshi, M. A. Bhat, Z. A. Kashoo, I. Hussain

**Affiliations:** Anaerobic Bacteriology Laboratory, Division of Veterinary Microbiology and Immunology, SKUAST-K, Shuhama (Alusteng), Srinagar, Jammu and Kashmir 190006 India

## Abstract

The aim of this study was to identify the immunodominant outer membrane proteins (OMPs) of *Fusobacterium necrophorum* from sheep affected with severe foot-rot. The OMP profile of ovine strains of *F. necrophorum* has not been well studied. We analyzed the OMP profile of the most frequent *lktA* variant JKS-F3 of *F. necrophorum* associated with severe ovine foot-rot with lesion score 4 in order to identify its major immunodominant OMPs. Electrophoretic separations of extracted OMPs showed a number of spots in two-dimensional electrophoretic gels. Two immunoreactive proteins of size around 43 kDa were identified through western blotting using hyperimmune sera raised in rabbits. These two immunogenic OMPs were analyzed by Matrix-assisted laser desorption/ionization time-of-flight mass spectrometry (MALDI-ToF/MS) which revealed that these two OMPs of *lktA* variant JKS-F3 of *F. necrophorum* showed 46 and 42 percent protein sequence coverage and scores of 125 and 114, respectively, with the reported 43 kDa outer membrane protein of *F. necrophorum* strain H05, a putative porin having properties similar to pore-forming proteins of anaerobic Gram-negative bacteria. These identified immunogenic OMPs will contribute to our understanding of the pathogenic role played by this organism in ovine foot-rot and could be exploited to devise an effective control strategy through development of an OMP-based recombinant vaccine to mitigate foot-rot in sheep and goats.

**Supplementary Information:**

The online version contains supplementary material available at 10.1007/s00284-021-02383-2.

## Introduction

*Fusobacterium necrophorum* is a Gram-negative, rod-shaped, non-spore-forming anaerobic bacterium that is associated with a variety of severe and fatal diseases in cattles, sheep and humans. In humans, *F. necrophorum* cause Lemierre syndrome, which begins as pharyngitis and rapidly progresses to septic thrombophlebitis of the jugular vein [1]. There are two main subspecies of *F. necrophorum*—ssp. *necrophorum* (Fnn-biotype A) and ssp. *fundiliforme* (Fnf-biotype B), and they differ in morphological, physiological and antigenic characteristics. Fnf is mostly implicated in human infection while Fnn being the principal animal pathogen mainly responsible for hepatic abscesses in beef cattle [2–4] and severe foot-rot in sheep and goats along with *Dichelobacter nodosus* [5, 6]. These anaerobes are more commonly detected in footrot affected than in healthy feet of sheep; hence, both need to be considered when managing a footrot outbreak [7].

Footrot begins as an inflammation of the interdigital skin of sheep and may progress to separation of hoof from the underlying soft tissues. The disease is an infectious syndrome, which is caused by the synergistic action of several bacterial species, particularly *D. nodosus* and *F. necrophorum* [8, 9]. *F. necrophorum* is an important pathogen contributing to the severity and duration of severe footrot (SFR) in sheep and goats and is often found in association with *D. nodosus* in infected feet with lesion score of mostly 4 [5, 6, 10].

Outer-membrane proteins (OMPs) of Gram-negative bacteria are integral proteins which assume a β-barrel architecture in the outer membrane and are arranged in an antiparallel pattern. OMPs are exposed to the outside of the bacterial cell and are the first line of contact between bacteria and its surroundings [11]. OMPs play many pivotal roles in pathogenesis, resistance and disease development by acting as a dynamic interface between the cell and its surroundings, maintenance of cell structure, involved in passive and active transport, adhesion to other cells, binding a variety of substances and antimicrobial resistance [12–14]. A 42.4 kDa outer membrane protein (FomA) of *F. n* ssp. *necrophorum* has been characterized and acts as an adhesin for strong binding to host cells, which is a key step in the disease pathogenesis [15]. However, at present there is no comprehensive information regarding the outer membrane proteins (OMPs) of *F. necrophorum* implicated in severe ovine foot-rot. Given the lack of information on the OMP profile of ovine strains of *F. necrophorum*, we identified and analyzed the OMPs of most predominant *lktA* variant JKS-F3 of *F. necrophorum* associated with severe foot-rot lesions in sheep by MALDI-ToF mass spectrometry. This information will further help in understanding the pathogenic mechanism of *F. necrophorum* in ovine footrot and adds to the list of target antigens that could be exploited for the development of an OMP-based recombinant vaccine to mitigate the severity of footrot lesions.

## Material and Methods

### Bacterial Culture and Identification of ***lktA***-Variant JKS-F3

*Fusobacterium necrophorum lktA* variant JKS-F3 was used in this study which is the most frequent variant (86.0%) detected in severe footrot-affected sheep with lesion scores of mostly 4 [6]. This variant was grown anaerobically on Brain–Heart–Infusion–Blood–Agar (BHIBA, Difco) containing 10% sheep blood, 0.5% yeast extract, 0.01% magnesium sulphate and antimicrobials, vancomycin and neomycin at 5 and 100 µg/ml of media, respectively. For large scale growth, isolated colonies of this variant were inoculated into freshly prepared 200 ml of BHI-broth containing all the above ingredients except sheep blood. The BHI-broth was then incubated anaerobically in a 1.5 liter anaerobic jar (Oxoid, UK) with AnaeroGaspack (Becton and Dickinson, Maryland, USA) at 37 °C for 48–72 h. The bacterial cells were pelleted by centrifugation at 6000×*g* for 20 min.

### DNA Etraction and Detection of *lktA* Gene Fragment

Few isolated colonies with characteristic morphology were directly suspended into 100 µl of nuclease free water in 1.5 ml microcentrifuge tubes and bacterial suspension was made by gentle vortexing. The suspension was boiled for 5 min, cooled on ice for 10 min and centrifuged at 10,000×*g* for 1 min. Two microlitres of the supernatant were used as the template for the PCR reaction. The DNA extract was subjected to the leukotoxin (*lktA*) gene-specific PCR for confirmation of variant JKS-F3 of *F. necrophorum* as detailed by [16]. The leukotoxin is the major virulence factor encoded by *lktA* gene of tricistronic leukotoxin operon (*lktBAC*) of *F. necrophorum*. The PCR conditions for amplification of *lktA* gene fragment consisted of initial denaturation at 94 °C for 4 min, followed by 35 cycles of 94 °C for 30 s, 60 °C for 30 s and 72 °C for 40 s. This was followed by final extension of 5 min at 72 °C. The DNA from *F. necrophorum* strain, which was isolated from severe ovine footrot and confirmed by DNA sequencing of its *lktA* gene fragment, served as positive control.

### Raising of Hyperimmune Sera in Rabbits

#### Preparation of Antigen

The JKS-F3 *lktA* variant was grown in BHI-broth as described. The bacterial cells were harvested and washed in PBS solution (pH 7.4) and killed with 0.15% formalin. To raise hyperimmune sera, approximately 10^8^ cells per dose per rabbit was used for immunization [17]. The density of the bacterial suspension was determined by matching it with turbidity standard (McFarland’s nephelometer).

#### Immunization of Rabbits

A New Zealand white rabbit of 1.5 kg weight was selected for immunization, and two doses 30 days apart were given. Prior to immunization, 2 ml of blood was collected to get the pre-immune sera. The first dose of antigen was prepared in Complete Freund’s Adjuvant in the ratio of 1:1 in a volume of 0.5 ml. The emulsion was prepared with the help of a syringe and a 18 gauge needle. The first dose was administered at four different sites subcutaneously, at 0.1 ml per site. The booster dose was prepared in a similar way but in Incomplete Freund’s Adjuvant. The blood was then collected at 7, 10, 14 and 21 days after the booster dose and the serum was separated. The antibody titer of the sera was checked through a micro-plate agglutination test, and the serum with high antibody titer was stored in aliquots at − 80 °C until use.

### Extraction of OMPs

For extraction of the OMPs, the bacterial cells were grown in bulk in BHI-broth as described above, harvested and washed in PBS. The harvested cells were suspended in 10 ml of 0.01 M HEPES buffer (pH 7.4), protease inhibitor cocktail was added to the suspension, which was subjected to ultrasonication in an ice-water at 30% amplitude with 15 pulses on/off of 59 s each. The sonicated cells were subjected to centrifugation at 17,000×*g* for 20 min to pellet the cell debris. The supernatant obtained was subjected to ultracentrifugation at 230,000*×*g for 70 min to pellet the membrane fraction. The pellet thus obtained was dissolved in 10 ml of 2% sodium sarcosinate in 0.01 M HEPES buffer (pH 7.4), incubated at room temperature for 60 min with gentle agitation and subjected to ultracentrifugation at 125,000×*g* for 110 min to get the pellet of OMPs. The pellet containing the OMPs was dissolved in 300–500 µl of sterile double distilled water and stored at − 80 °C until use.

### Estimation and Ceaning Up of OMPs for 2-Dimensional Electrophoresis (2DE)

The protein estimation and cleaning up of the samples was performed using the 2-D Quant Kit and 2-D Cleanup Kit (GE Healthcare), respectively, according to the manufacturer’s instructions.

For 2DE, briefly,13-cm immobiline DryStrip gels (IPG strip, GE Healthcare) with a pH range 3–11 were hydrated over night with 250 µl of extracted OMPs (600 µg/strip) resuspended in DeStreak rehydration solution in the Immobiline DrySrip re-swelling trays (GE Healthcare) at room temperature. Following complete hydration of the strips, first dimension isoelectric focusing (IEF) was performed in a Ettan IPG phorII (GE Healthcare) with an appropriate software for 3–11 pH range and 13-cm-long Immobiline DryStrips. After IEF, the IPG strips were equilibrated for the second dimension. The equilibration was performed by first treating strips with dithiothreitol (100 mg) and then with iodoacetamide (250 mg) per 10 ml of equilibration buffer separately for 15 min each with gentle shaking.

After IEF and strip equilibration, SDS-PAGE was performed in an SE 600 Ruby vertical electrophoresis unit (GE Healthcare) using a 12.5% polyacrylamide gel. The conditions for the runs were: 430 V, 120 mA and 100 W for two gels loaded simultaneously. After completion of the run, one gel was subjected to staining with Pulse one Coomassie PhastGel Blue R-350 (GE Healthcare) for visualization of OMPs and the other was subjected to western blotting.

### Western Blot Aalysis of OMPs with Sra from Rabbit

The gel from the second-dimension SDS-PAGE was blotted onto polyvinylidene difluoride (PVDF) (Hybond-P, GE Healthcare) membrane for detection of immunoreactive OMPs. The conditions used for transfer were 30 V maximum, 430 mA current for about 2 h. After the protein transfer, the PVDF membranes were blocked with 5% non-fat skimmed milk in PBS at room temperature on a rocker overnight. After washing three times with 0.1% Tween-20 in PBS(PBST) for 10 min each, the PVDF membrane was incubated with primary antibody raised in rabbit (as above) at a dilution of 1:1000 in PBS containing 1% Bovine Serum Albumin for 1.30 h at room temperature on a rocker. The membrane was washed in PBST three times, 10 min each and incubated with secondary antibody (anti-rabbit IgG raised in goat and conjugated with HRPO, Sigma) at a dilution of 1:3000 in PBS for 1.30 h, with gentle shaking on a rocker at room temperature. The membrane was again washed three times with PBST, 10 min each and developed with 0.05% 3,3′-diaminobenzidine solution until protein spots were visualized.

#### Identification of Immunoreactive OMPs

The protein spots (OMPs) from the stained gel corresponding to the developed spots on the PVDF membrane were picked and stored in 8% acetic acid solution and outsourced for MALDI-TOF/MS analysis for protein identification.

## Results

### Isolation and Detection of JKS-F3 *lktA* Variant of *F. necrophorum*

**Fig. 1:**
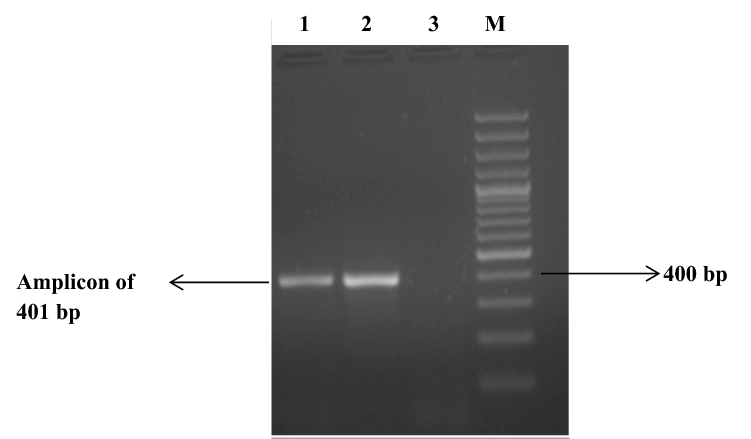
Detection of *lktA* gene fragment of variant JKS-F3 of *F. necrophorum* by polymerase chain reaction. Lane 1 = Test sample. Lane 2 = Positive control. Lane 3 = Negative control. Lane M = 100 bp DNA ladder

After subsequent subculture of JKS-F3 variant on BHIBA containing 10% sheep blood, pure culture of *F. necrophorum* was obtained. The colonies on BHIBA appeared as flat, irregular, grayish and surrounded by zone of *β*-haemolysis and produced characteristic lipolytic activity on egg yolk agar medium. The lipolytic activity is due to the lipase produced by bacteria that hydrolyze free fat present in the medium to glycerol and free fatty acids. Insoluble free fatty acids results in the development of an iridescent sheen (as with oil on water) that can be seen when the plate is held at an angle to a light source. The Gram-stained smear demonstrated pleomorphic character of *F. necrophorum.* For further confirmation, isolated colonies were subjected to PCR targeting 400 base pair (bp) amplicon, characteristic of the *lktA* gene fragment of *F. necrophorum* (Fig. 1).

### OMP Profile of JKS-F3 *lktA* Variant of *F. necrophorum*

**Fig. 2:**
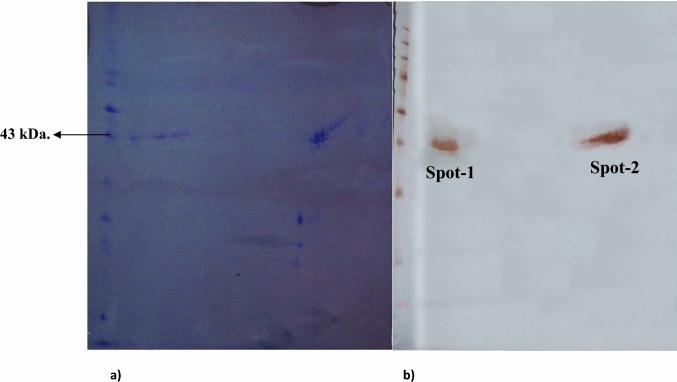
2DE profile of OMP fraction of variant JKS-F3 of *F. necrophorum* (**a**) and its western blot on PVDF membrane (**b**)

A number of protein spots ranging from 29 to 54 kDa were visualized following two-dimensional gel electrophoretic separations of OMP extracts of a JKS-F3 *lktA* variant of *F. necrophorum.* Based on the SDS–PAGE analysis, a few protein spots of sizes around 43 kDa were more prominent than the other spots (Fig. 2a). Western blotting revealed only two prominent protein spots (Spot 1 and Spot 2) on the PVDF membrane using primary antibodies raised in rabbit against the JKS-F3 *lktA* variant of *F. necrophorum* (Fig. 2b). These two major immunoreactive spots both correspond to molecular weight of around 43 kDa with distinctly separated isoelectric points on the 13 cm immobiline DryStrip gel with a pH range 3–11.

### Identification of Immunodominant OMPs of *F. necrophorum* By MALDI-ToF Mass Spectrometr*y*

**Fig. 3:**
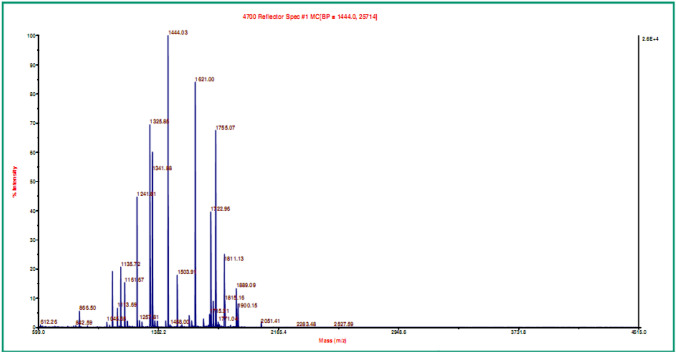
Peptide mass fingerprint of spot-1 after tryptic digestion

**Fig. 4:**
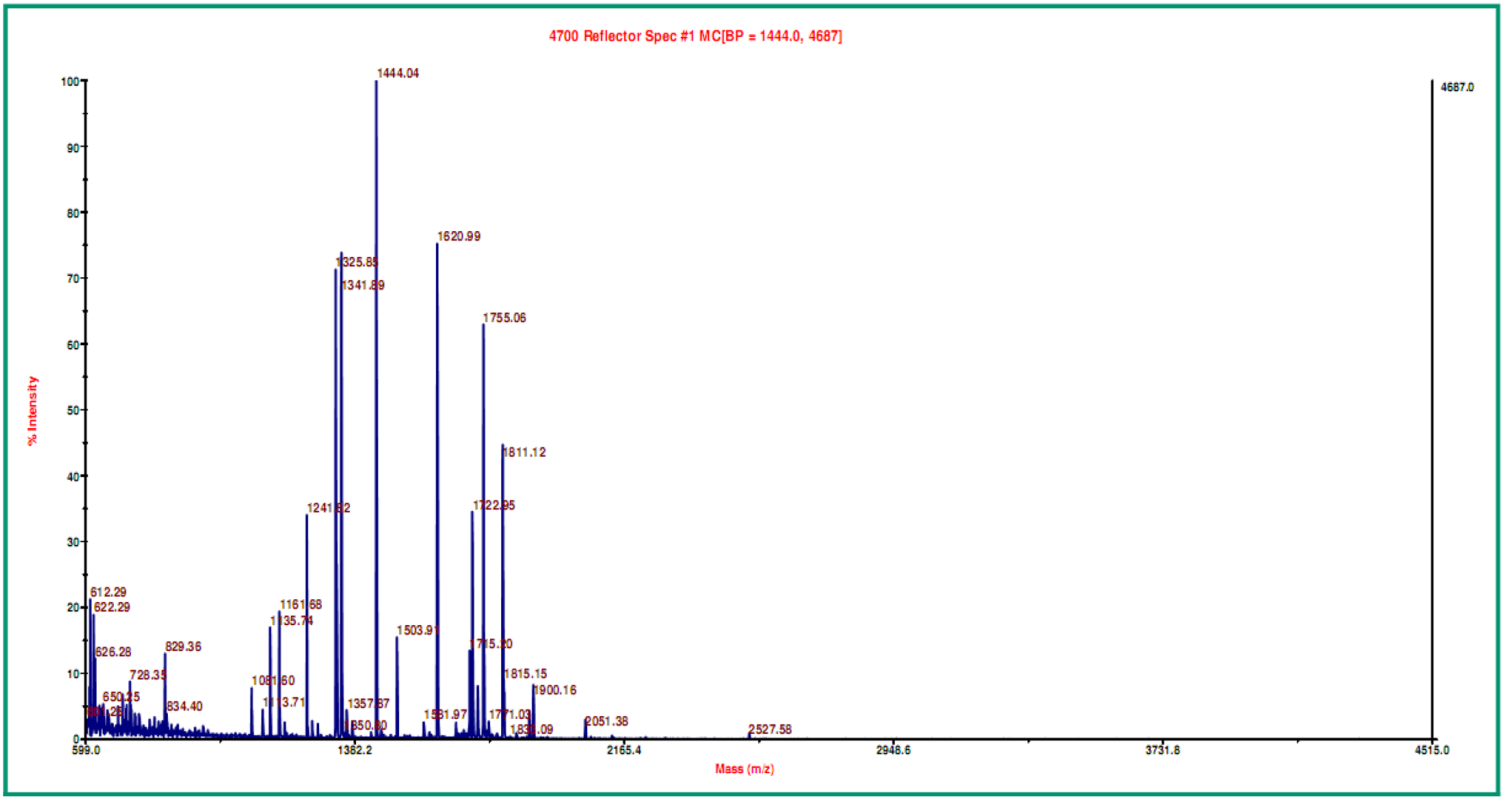
Peptide mass fingerprint of spot-2 after tryptic digestion

**Fig. 5:**
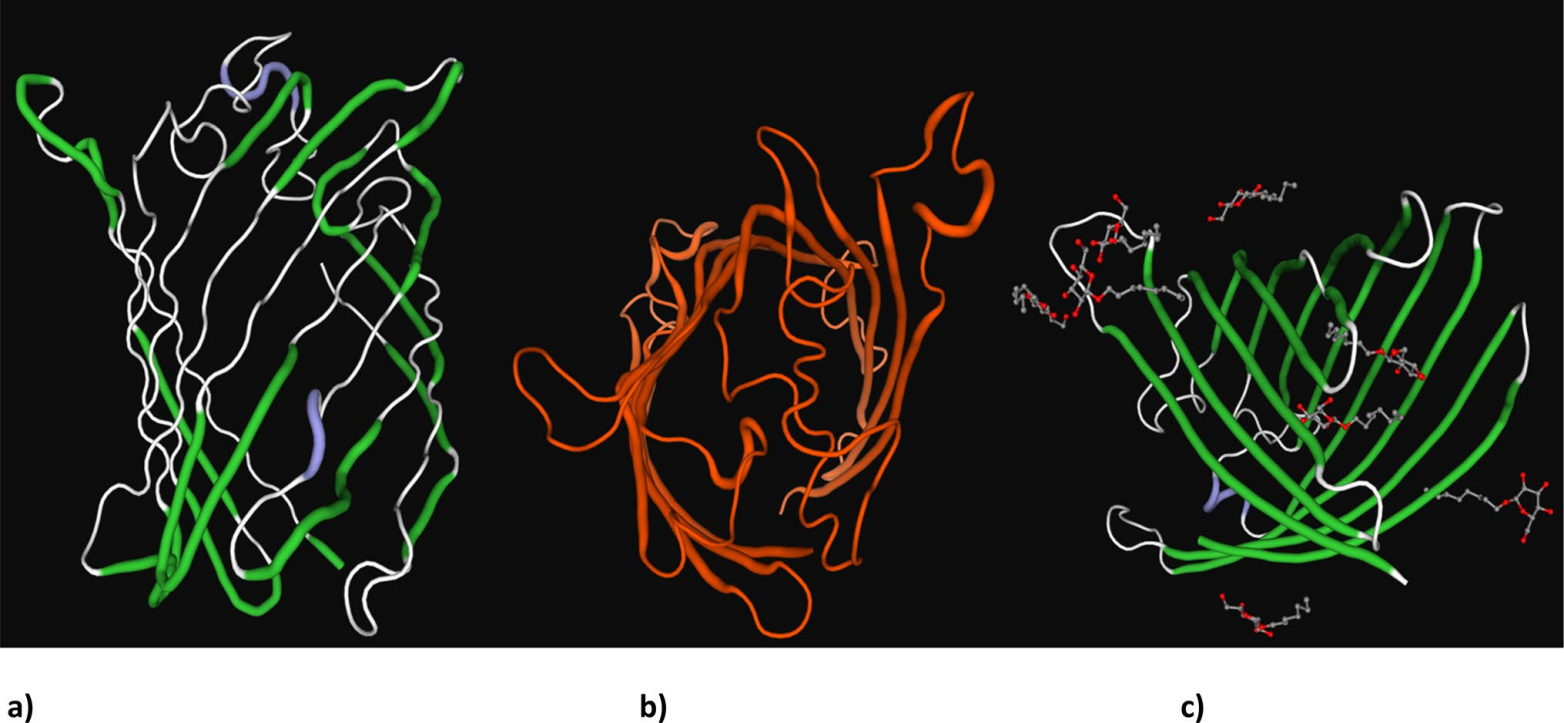
The predicted 3D structure of 43 kDa OMP (porin) of *F. necrophorum* strain H05 depicting front (**a**), top (**b**) and lateral (**c**) views, respectively

The peptide mass fingerprints (PMFs) generated by MALDI-ToF MS after tryptic digestion of OMPs produced sixteen and fifteen peptides for spots 1 and 2, respectively. The PMF data obtained for these unknown OMPs were analyzed by matching with the PMFs of *F. necrophorum* proteome contained in NCBI protein database using the MASCOT search results. The results depicted top ten protein matches for each OMP along with their accession numbers, mass, protein sequence coverage, score and description. The OMPs obtained from *lktA* variant JKS-F3 of *F. necrophorum* showed highest sequence coverage (46 and 42%) and scores (125 and 114) with the reported 43 kDa OMP, a putative porin protein of *F. necrophorum* strain H05 (Accession No. AFJ54023.1) (Figs. 3, 4). The protein scores greater than 84 are considered significant with *p* value <0.05. The identified OMPs were homologous with OMP of *F. necrophorum* strain H05 consisting of 377 amino acid residues. Its 3D structure was built in ExPASy swiss-model which revealed that its structure resembles porin proteins of outer membrane (Fig. 5).

## Discussion

The *Fusobacterium* is a genus-containing anaerobic bacteria and comprises of several species, in which *F. necrophorum* is a key human and animal pathogen and is most frequently isolated from necrotic infections [18]. Like other Gram-negative bacteria, outer membrane proteins (OMPs) of *F. necrophorum* are important in facilitating attachment and is the first bacterial component to come in contact with the host cell surface and hence play a vital role in establishment of infection and virulence [19]. OMPs are diverse in nature and play numerous roles such as adhesins, porins, acts as channels for the uptake of nutrients and ions, function as receptors for phages, etc. [11].

In the present study, two immunogenic OMPs from JKS-F3 *lktA* variant of *F. necrophorum* were identified by MALDI-ToF mass spectrometry. Identification of microbes or its proteins by MALDI-TOF MS is done by either comparing the PMF of unknown organism/protein with the PMFs contained in the database, or by matching the masses of biomarkers of unknown organism with the proteome database [20]. MALDI-ToF MS analysis of *F. necrophorum* OMPs revealed that these are homologous with 43 kDa OMP of *F. necrophorum* strain H05, a putative porin on the basis of structure, protein sequence coverage and significant protein score. This putative porin has similar properties as that of other pore-forming proteins of Gram-negative anaerobic bacteria [21]. The predicted 3D structure of this OMP, containing 377 amino acid residues was built in ExPASy swiss-model which showed that it is rich in *β*-barrel strands forming channel across the bacterial outer membrane. Porins are a class of immunogens that are membrane-associated integral proteins and form channels which facilitate the diffusion of small water-soluble molecules across bacterial outer membrane and contribute to virulence by interacting with host cells. Porins possess a high proportion of *β*-sheet structure, which traverses the membrane in a tightly packed *β*-barrel organization. This is in agreement with the findings of [15] who successfully cloned and expressed the 42.4 kDa OMP (*fom A* gene) from *F. necrophorum* ssp. *necrophorum* in *E. coli* and showed that it increases the binding of *E. coli* to bovine adrenal gland capillary endothelial (EJG) cells, compared to the un-induced or control vector expressed in *E. coli* and concluded that the 42.4 kDa OMP serves as an adhesin for endothelial cells for bovine strains of *F. necrophorum* and may play an important role in host colonization and pathogenesis. Similarly [19], studied the OMP profiles of two sub-species of *F. necrophorum* and concluded that sub-species *necrophorum* and *funduliforme* had a 40 and 37.5 kDa immunodominant OMP, respectively, which is in agreement with the present findings.

In the present study, identification of two immunogenic OMPs by 2DE-MALDI-ToF/MS from a frequently encountered JKS-F3 *lktA* variant of *F. necrophorum* adds to the list of potential target antigens and should be of great interest in terms of understanding the host–pathogen interactions. These immunogenic proteins could be exploited for the development of OMP-based recombinant vaccine to mitigate severe foot-rot in sheep and goats.

## Conclusion

The present investigation identified two immunodominant OMPs from JKS-F3 *lktA* variant of *F. necrophorum.* This variant is frequently detected in severe ovine footrot with lesion score 4 indicating that it likely contributes to the severity and duration of the ovine footrot lesions. These immunodominant OMPs could be exploited for the production of OMP-based recombinant vaccine against footrot to mitigate its severity.

## Supplementary Information

Below is the link to the electronic supplementary material.

Supplementary file 1 (PPTX 1.94 MB)
