# Supplementary material for: Identification of Immunodominant Outer Membrane Proteins of Fusobacterium necrophorum from Severe Ovine Footrot By MALDI-TOF Mass Spectrometry
Source: Curr Microbiol. 2021 Feb 27;78(4):1298–304. doi: 10.1007/s00284-021-02383-2 (PMC7997824; doi:10.1007/s00284-021-02383-2)
Supplement: Supplementary file 1 — (PPTX 1.94 MB) [file 284_2021_2383_MOESM1_ESM.pptx]

## Slide 1
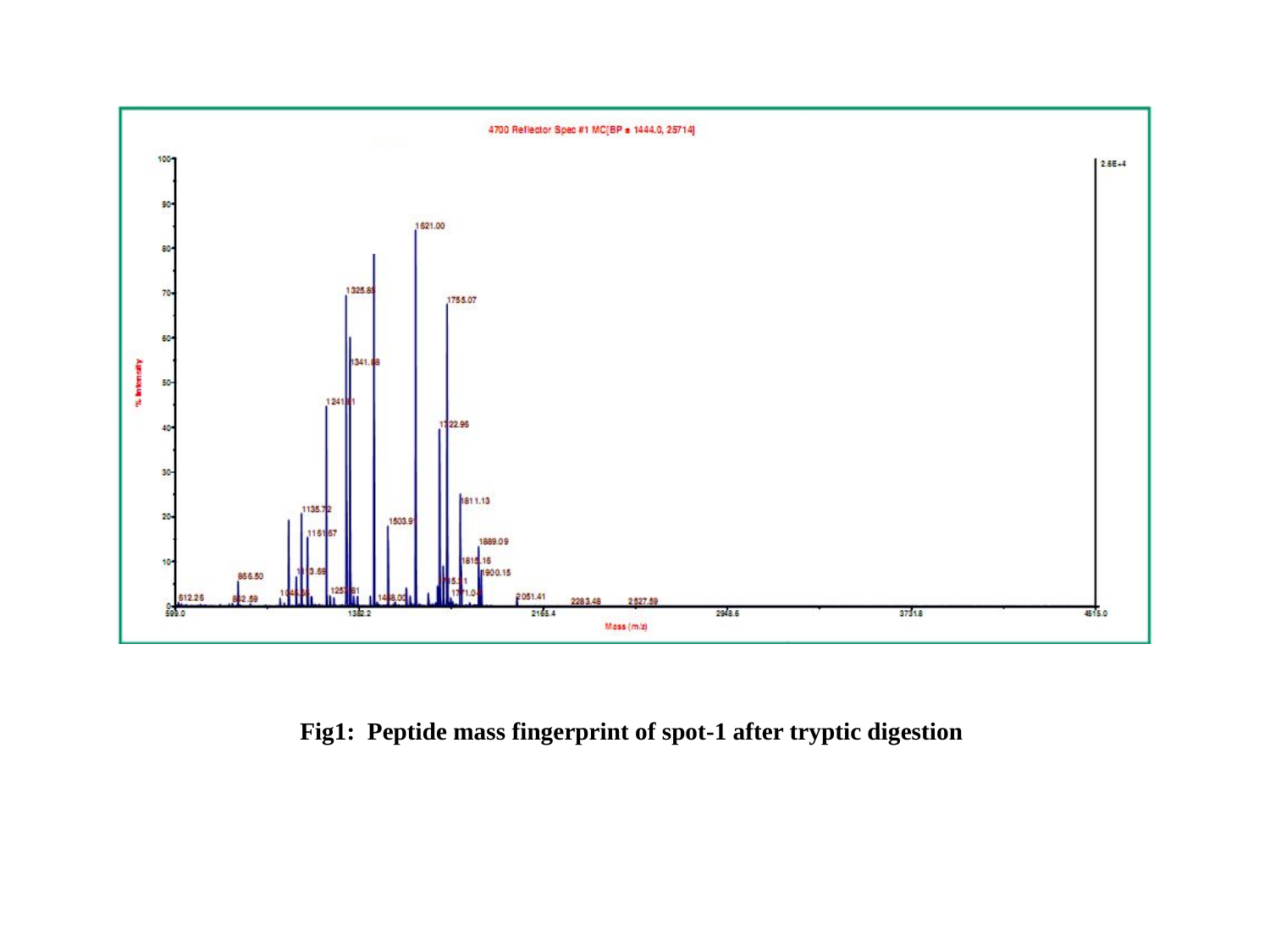

Fig1: Peptide mass fingerprint of spot-1 after tryptic digestion

## Slide 2
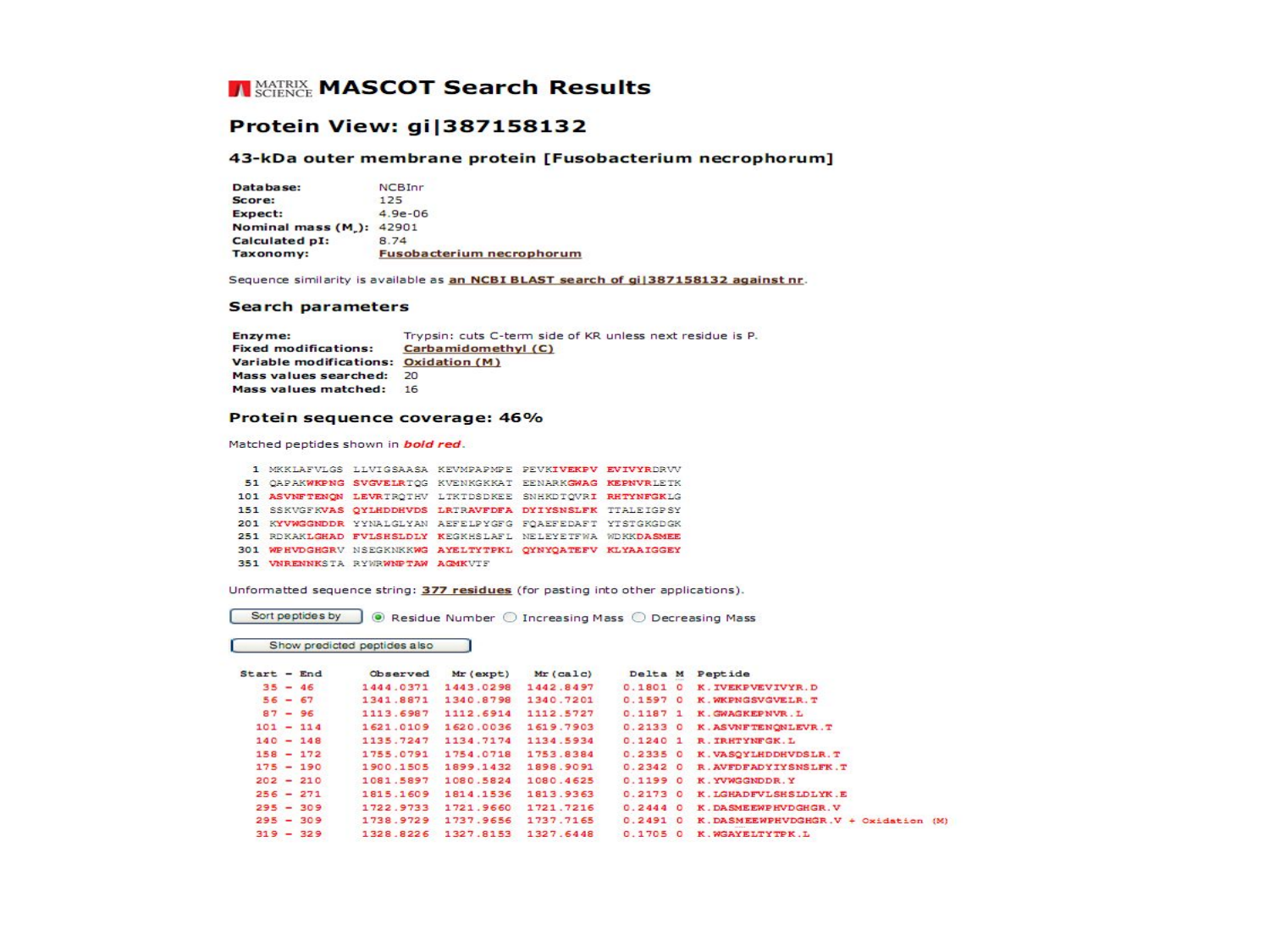

## Slide 3
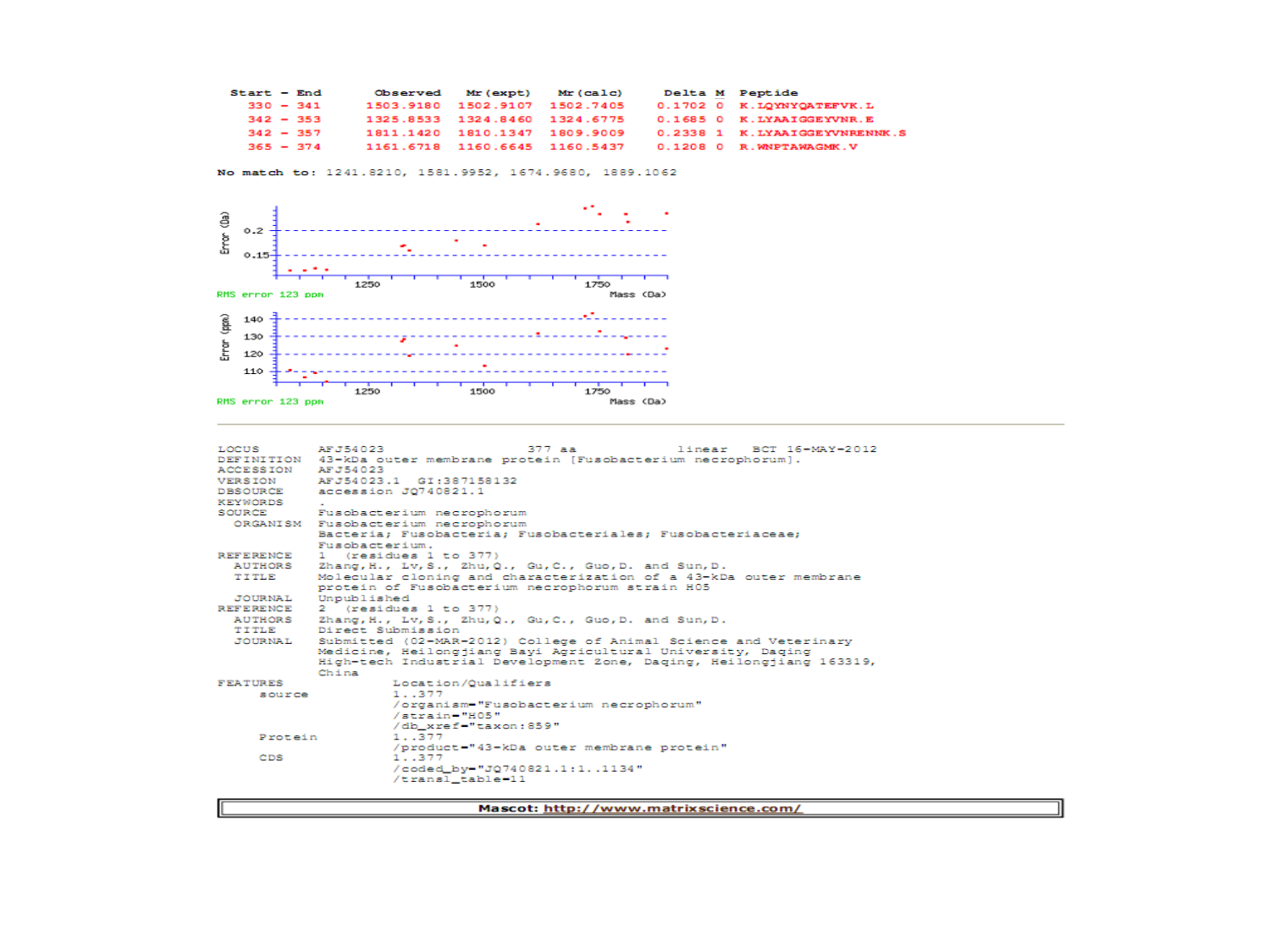

## Slide 4
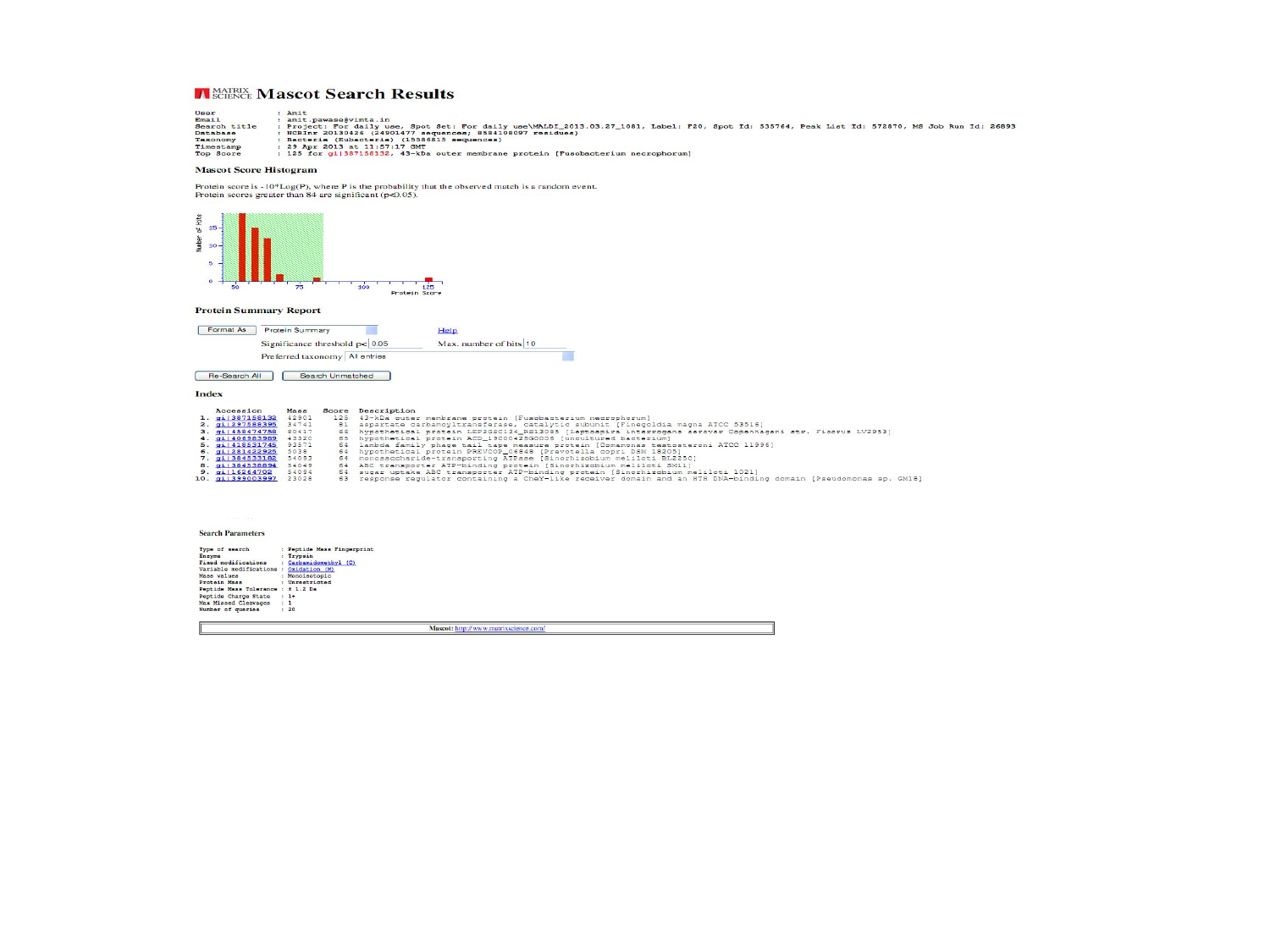

## Slide 5
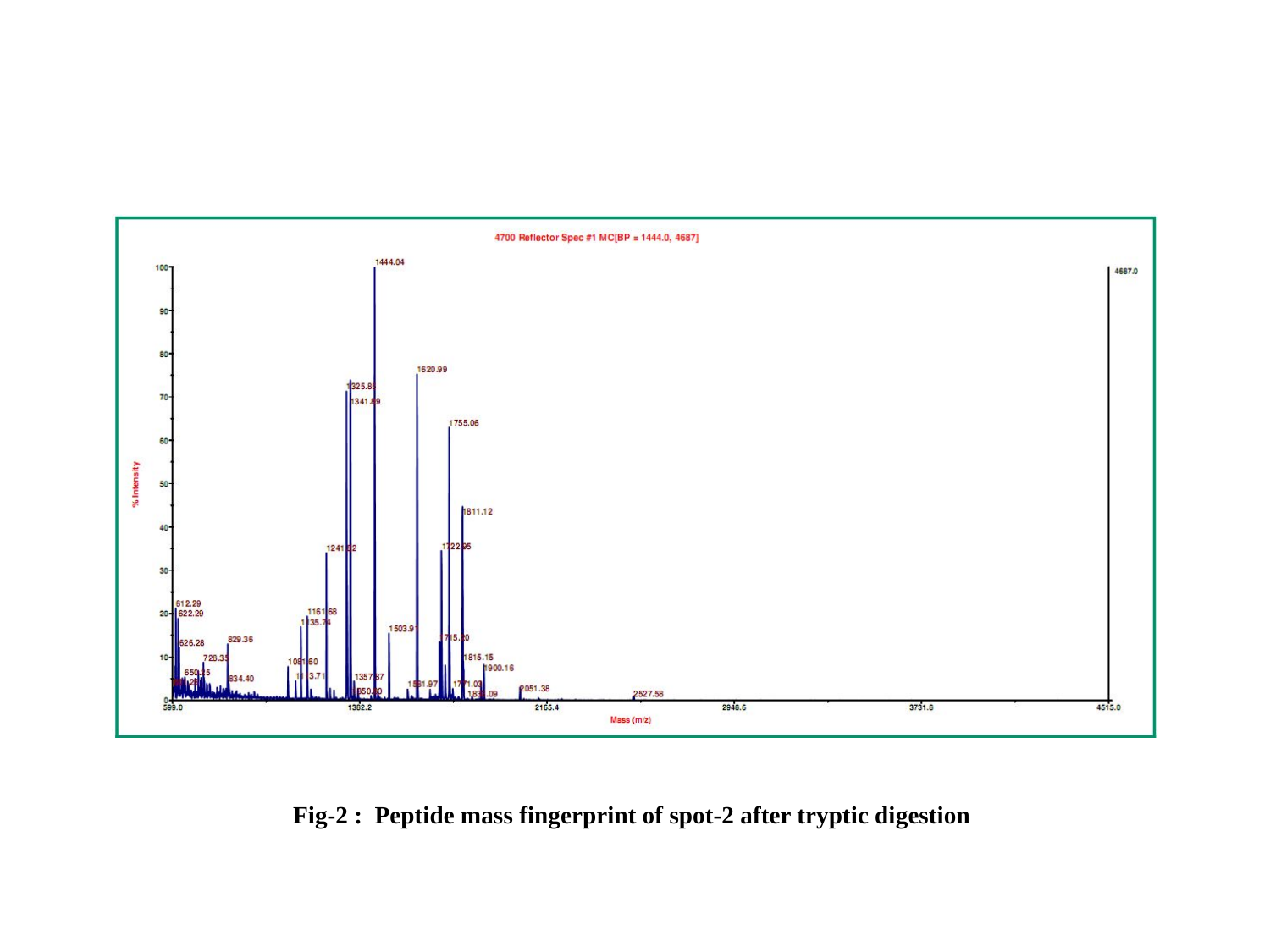

Fig-2 : Peptide mass fingerprint of spot-2 after tryptic digestion

## Slide 6
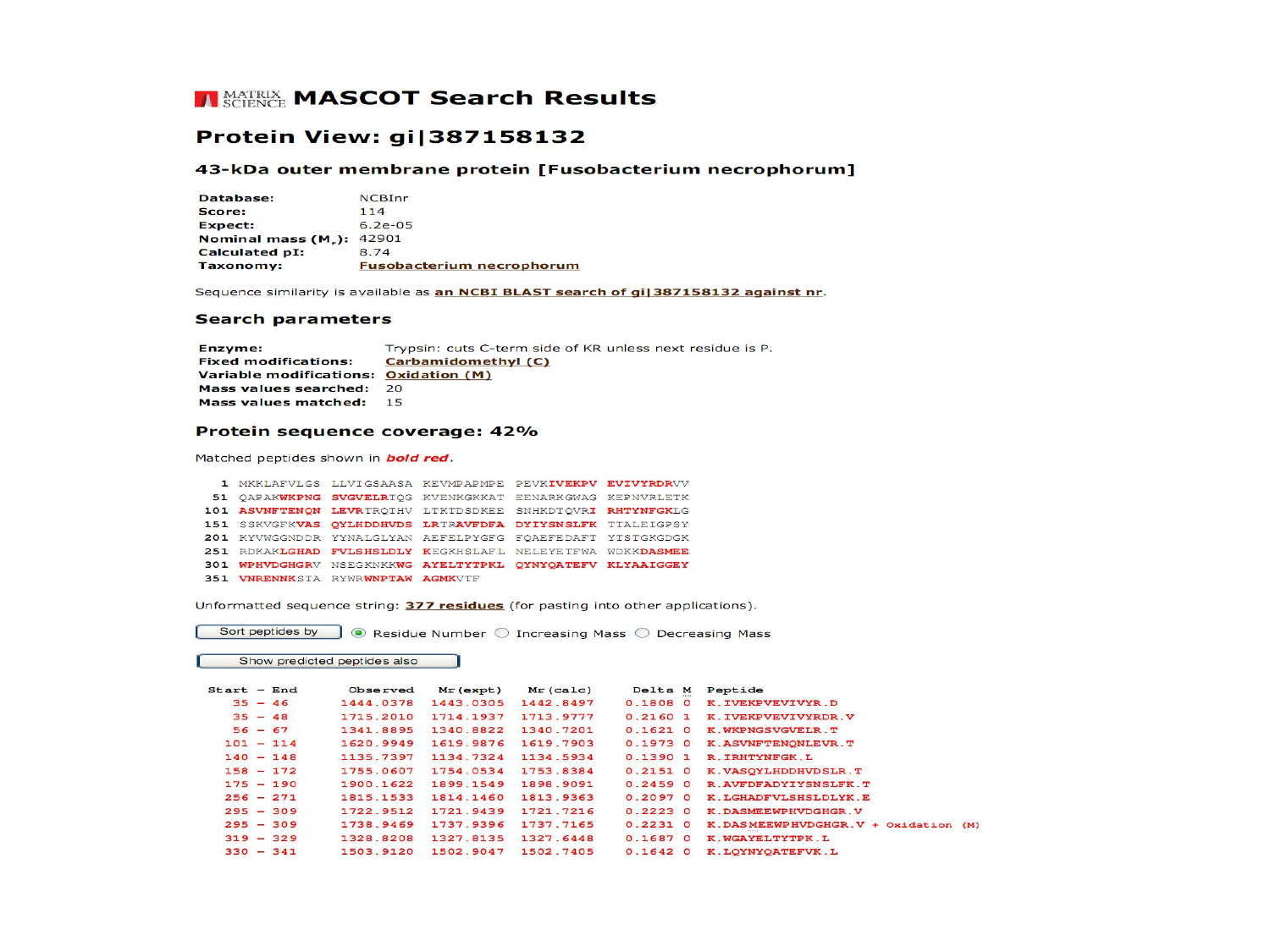

## Slide 7
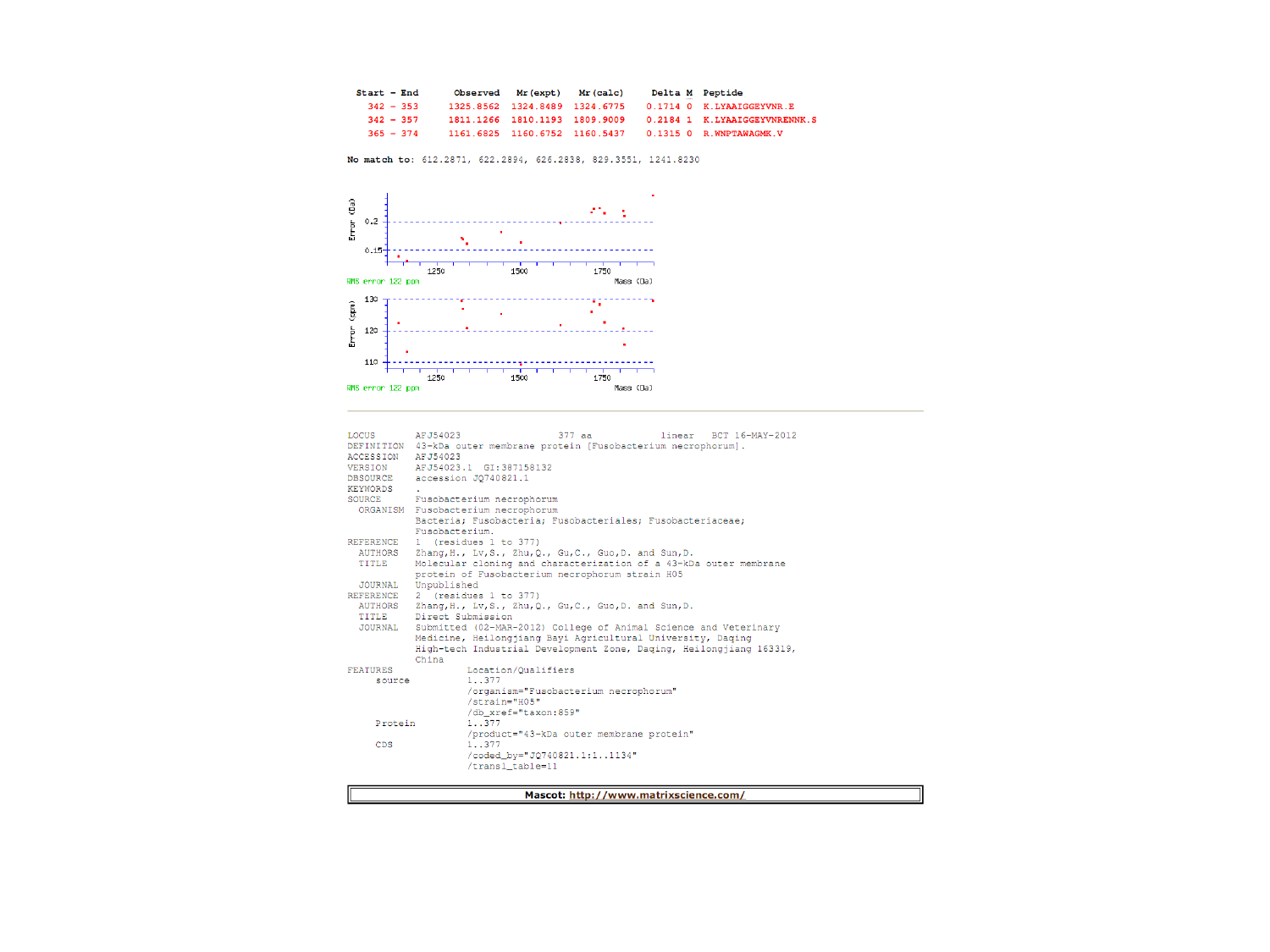

## Slide 8
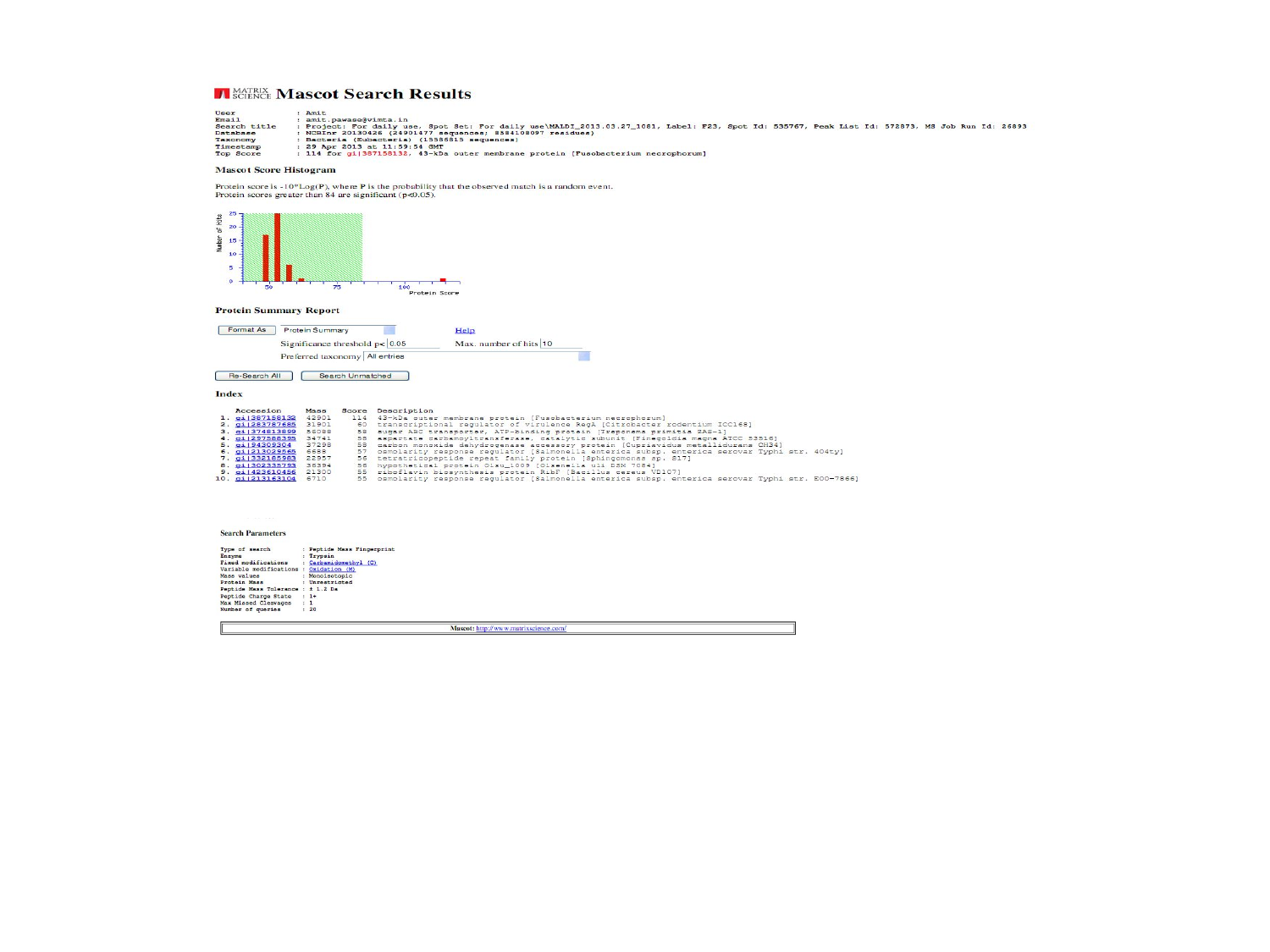

## Slide 9
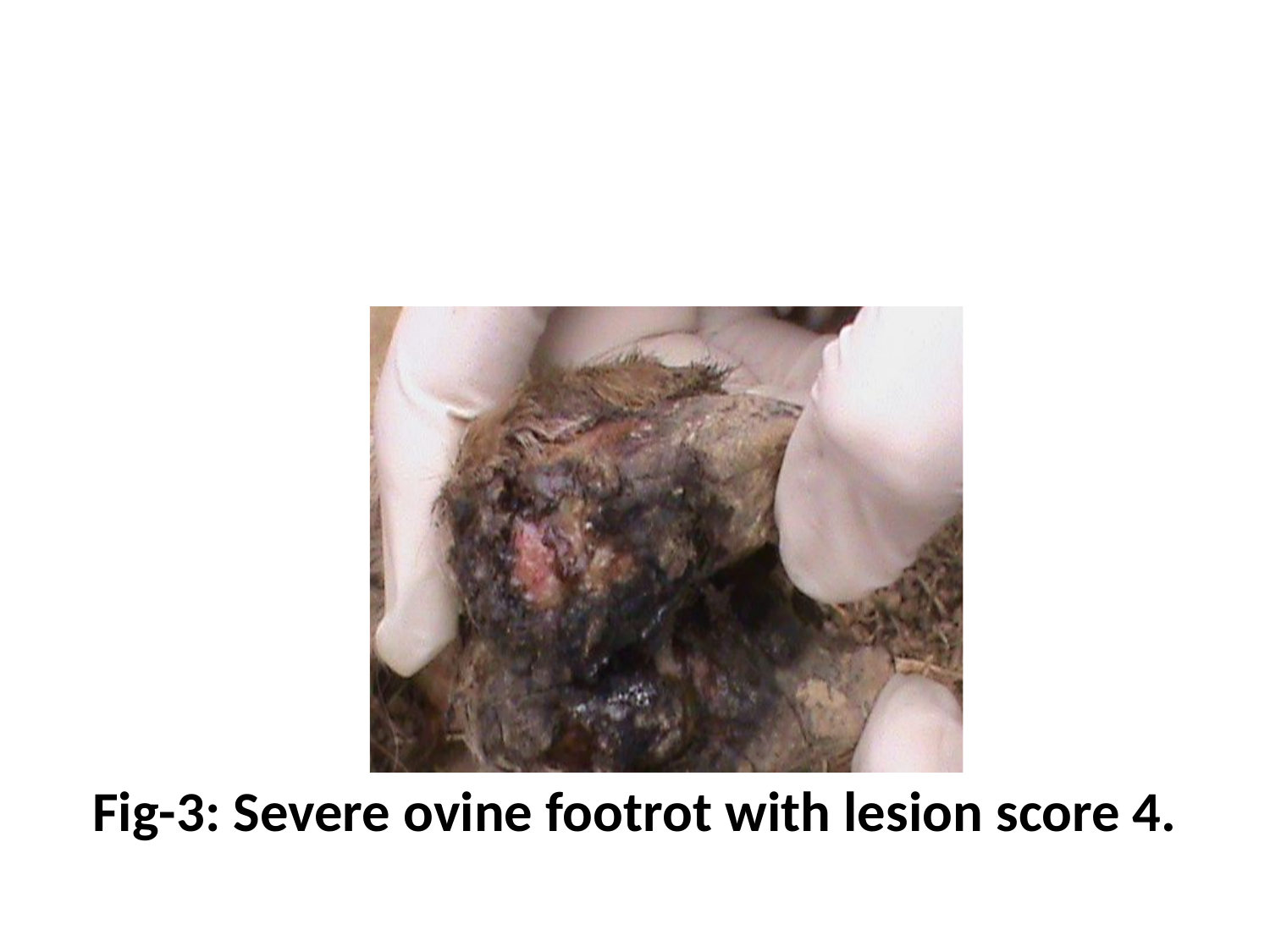

# Fig-3: Severe ovine footrot with lesion score 4.

## Slide 10
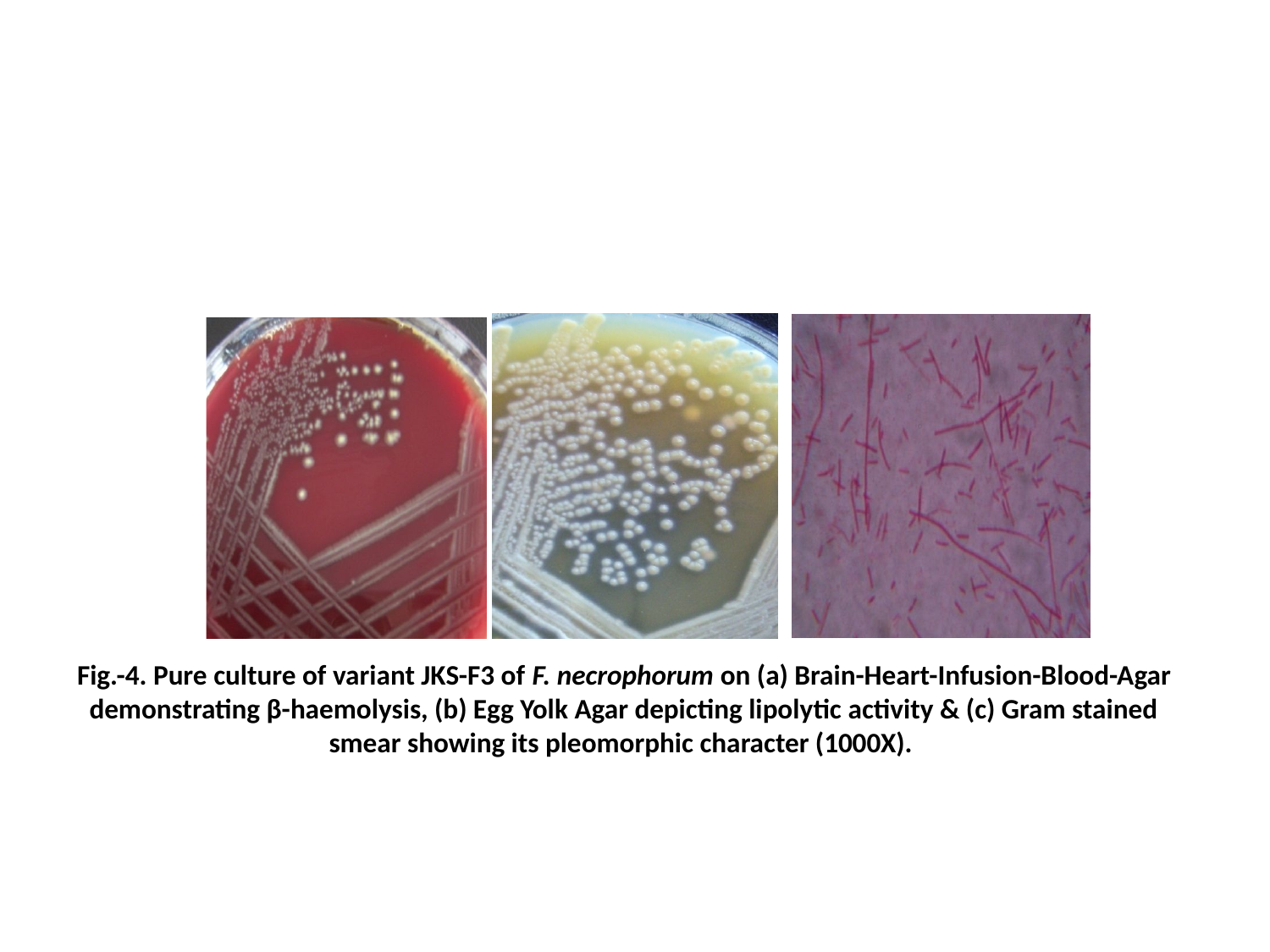

# Fig.-4. Pure culture of variant JKS-F3 of F. necrophorum on (a) Brain-Heart-Infusion-Blood-Agar demonstrating β-haemolysis, (b) Egg Yolk Agar depicting lipolytic activity & (c) Gram stained smear showing its pleomorphic character (1000X).

## Slide 11
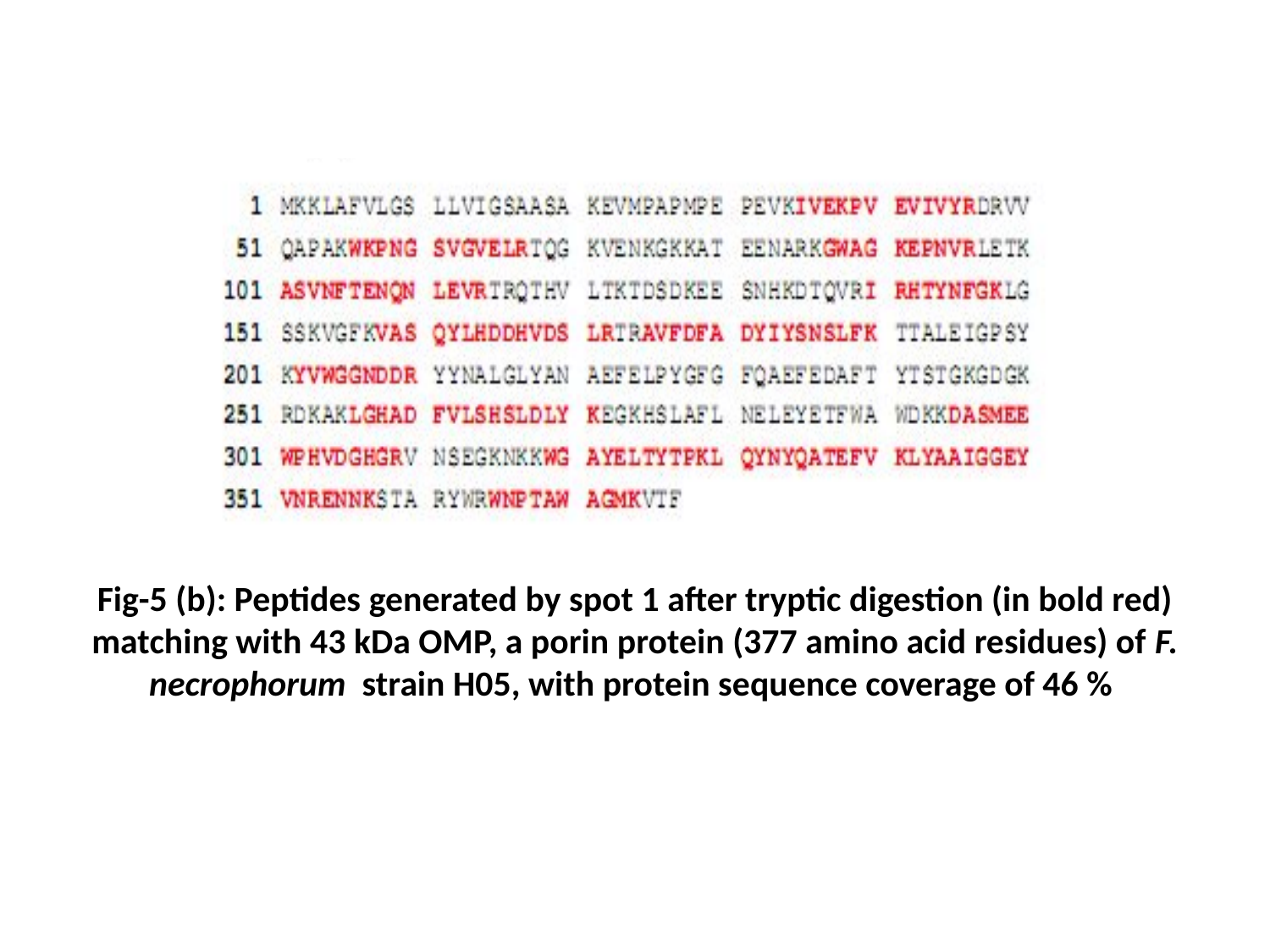

# Fig-5 (b): Peptides generated by spot 1 after tryptic digestion (in bold red) matching with 43 kDa OMP, a porin protein (377 amino acid residues) of F. necrophorum strain H05, with protein sequence coverage of 46 %

## Slide 12
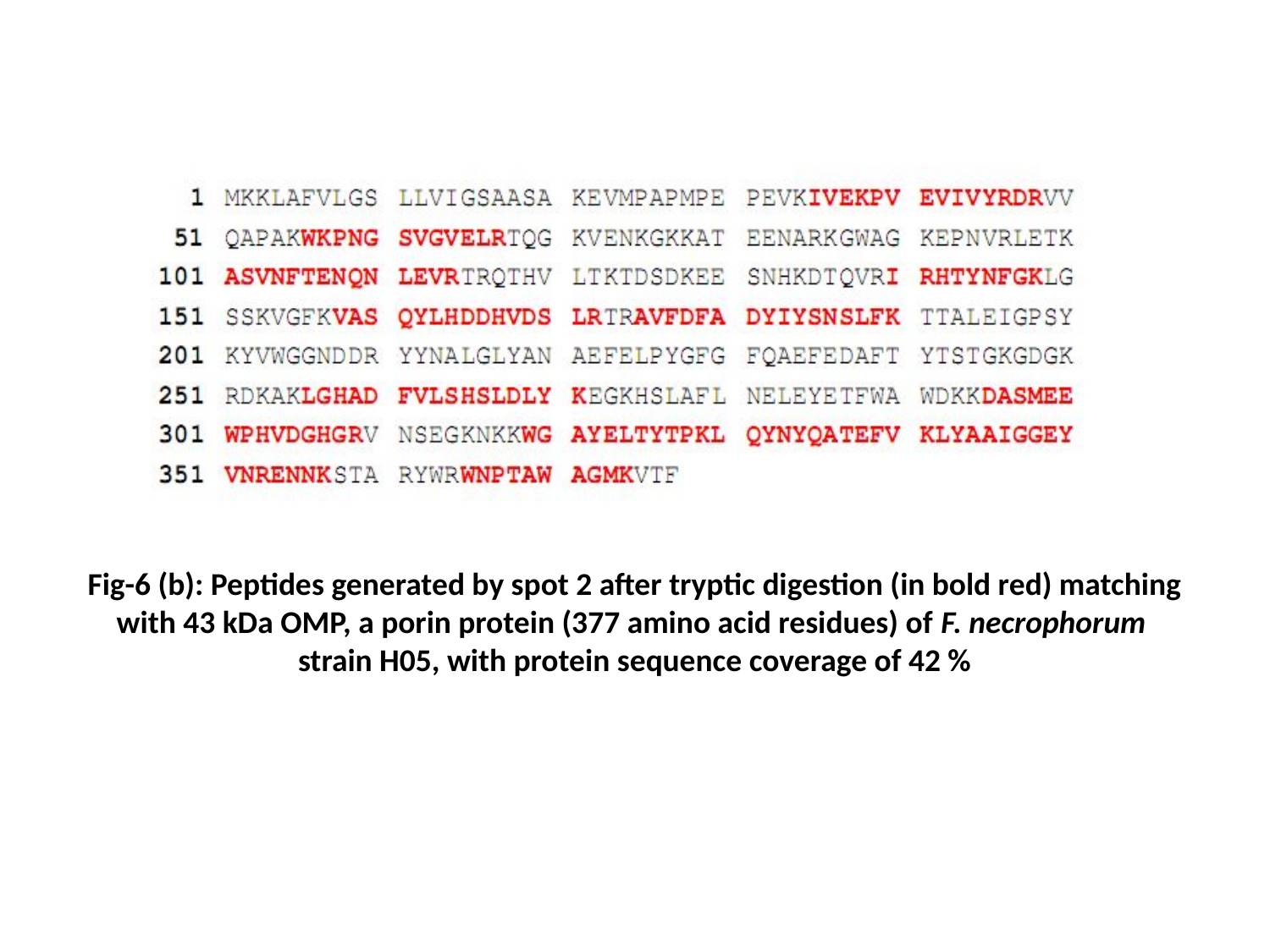

# Fig-6 (b): Peptides generated by spot 2 after tryptic digestion (in bold red) matching with 43 kDa OMP, a porin protein (377 amino acid residues) of F. necrophorum strain H05, with protein sequence coverage of 42 %
